# A complete landscape of post-transcriptional modifications in mammalian mitochondrial tRNAs

**DOI:** 10.1093/nar/gku390

**Published:** 2014-05-15

**Authors:** Takeo Suzuki, Tsutomu Suzuki

**Affiliations:** Department of Chemistry and Biotechnology, Graduate School of Engineering, University of Tokyo, Bunkyo-ku, Tokyo 113-8656, Japan

## Abstract

In mammalian mitochondria, 22 species of tRNAs encoded in mitochondrial DNA play crucial roles in the translation of 13 essential subunits of the respiratory chain complexes involved in oxidative phosphorylation. Following transcription, mitochondrial tRNAs are modified by nuclear-encoded tRNA-modifying enzymes. These modifications are required for the proper functioning of mitochondrial tRNAs (mt tRNAs), and the absence of these modifications can cause pathological consequences. To date, however, the information available about these modifications has been incomplete. To address this issue, we isolated all 22 species of mt tRNAs from bovine liver and comprehensively determined the post-transcriptional modifications in each tRNA by mass spectrometry. Here, we describe the primary structures with post-transcriptional modifications of seven species of mt tRNAs which were previously uncharacterized, and provide revised information regarding base modifications in five other mt tRNAs. In the complete set of bovine mt tRNAs, we found 15 species of modified nucleosides at 118 positions (7.48% of total bases). This result provides insight into the molecular mechanisms underlying the decoding system in mammalian mitochondria and enables prediction of candidate tRNA-modifying enzymes responsible for each modification of mt tRNAs.

## INTRODUCTION

Mitochondria, organelles present in most eukaryotic cells, provide the chemical energy required by living cells in the form of adenosine triphosphate (ATP), which is synthesized by the electron transport chain and oxidative phosphorylation (1). Mitochondria contain their own genomic DNA, called mitochondrial (mt)DNA, and unique transcription and translation machinery that converts their genetic information into proteins. In mammals, the mtDNA is a circular double-stranded DNA, ∼16 kilobase pairs (kb) in length, which contains 13 genes encoding essential subunits of the respiratory chain complexes and 24 RNA genes (2 ribosomal RNAs and 22 tRNAs) required for mitochondrial protein synthesis.

The mammalian mitochondrial decoding system differs from the canonical decoding system by its use of four non-universal codons (Table 1) (2): AUA for Met, UGA for Trp and AGR (R = A or G) for Stop. The 60-sense codons are deciphered by 22 species of mitochondrial tRNAs, which constitute the smallest set of tRNAs necessary to translate all sense codons among all kingdoms of life, including other organelle decoding systems. Post-transcriptional modifications at the first letters of tRNA anticodons play a critical role in establishing this minimal decoding system. To reduce the number of tRNA species, each of eight family boxes in mitochondria is decoded by only a single tRNA. The tRNAs responsible for the family boxes frequently have unmodified uridines (U34) at the first (wobble) position of an anticodon. According to Crick's wobble rule (3), U34 can recognize only A and G at the third position of a codon. In the decoding systems of some bacteria and most organelles, U34 can base-pair with any of the four bases by the so-called ‘four-way wobble rule’ (2) or ‘super wobbling’ (4). The conformational flexibility of U34 is thought to enable U:U and U:C pairing. On the other hand, tRNAs responsible for two-codon sets ending in purines (NNR; N = any four nucleotides) have modified uridines (xm^5^s^2^U-type) at their wobble positions (5). In general, xm^5^s^2^U-type modification restricts conformational flexibility of the wobble base, thereby strengthening recognition of NNR codons and preventing misrecognition of codons ending in pyrimidines (NNY; Y = U and C). We previously discovered that taurine-containing modified uridines in mammalian mt tRNAs are responsible for NNR codon sets: 5-taurinomethyluridine (τm^5^U) at the wobble position of human and bovine mt tRNA^Leu(UUR)^ (6), and 5-taurinomethyl-2-thiouridine (τm^5^s^2^U) at the wobble position of human and bovine mt tRNA^Lys^ (6). Subsequently, τm^5^s^2^U was also found at the wobble position of the bovine mt tRNAs for Glu and Gln (7). Determining the proper usage of unmodified or modified U34 in tRNAs would reveal a fundamental principle of the minimal decoding system in mammalian mitochondria.

**Table 1.** Codon–anticodon pairing in the bovine mitochondrial genetic code.

Codon	Amino acid (anticodon)	Codon	Amino acid (anticodon)	Codon	Amino acid (anticodon)	Codon	Amino acid (anticodon)
UUU	Phe	UCU		UAU	Tyr	UGU	Cys
UUC	(GAA)	UCC	Ser	UAC	(QUA)	UGC	(GCA)
UUA	Leu	UCA	(UGA)	UAA	stop	**UGA**	Trp
UUG	(τm^5^UAA)	UCG		UAG		UGG	(τm^5^UCA)
CUU		CCU		CAU	His	CGU	
CUC	Leu	CCC	Pro	CAC	(QUG)	CGC	Arg
CUA	(UAG)	CCA	(UGG)	CAA	Gln	CGA	(UCG)
CUG		CCG		CAG	(τm^5^s^2^UUG)	CGG	
AUU	Ile	ACU		AAU	Asn	AGU	Ser
AUC	(GAU)	ACC	Thr	AAC	(QUU)	AGC	(GCU)
**AUA**	Met	ACA	(UGU)	AAA	Lys	**AGA**	stop
AUG	(f^5^CAU)	ACG		AAG	(τm^5^s^2^UUU)	**AGG**	
GUU		GCU		GAU	Asp	GGU	
GUC	Val	GCC	Ala	GAC	(QUC)	GGC	Gly
GUA	(UAC)	GCA	(UGC)	GAA	Glu	GGA	(UCC)
GUG		GCG		GAG	(τm^5^s^2^UUC)	GGG	

Non-universal genetic codes are denoted in bold type. AUA: Ile (universal), Met (mitochondria); UGA: stop (universal), Trp (mitochondria); AGA/G: Arg (universal), stop (mitochondria).

5-formylcytidine (f^5^C), another unique modification in mammalian mitochondria (8), is present at the wobble position of mt tRNA^Met^. Biochemical studies show that f^5^C is required for recognition of the non-universal AUA codon, in addition to the canonical AUG codon (9,10). Possible base pairing between f^5^C and A was demonstrated by a crystallographic study using an f^5^C-containing anticodon stem-loop bound to the 30S subunit of *Thermus thermophilus* (11).

Defective mitochondrial translation results in mitochondrial dysfunction, ultimately causing pathological consequences (12). Numerous pathogenic mutations associated with mitochondrial diseases have been found in mtDNA (http://www.mitomap.org/MITOMAP) (13). These pathogenic mutations are maternally inherited. Among over 400 pathogenic mutations compiled to date, ∼200 have been mapped to mt tRNA genes; these mutations diminish biogenesis, stability and function of tRNAs (2,12,14–15). Two major subgroups of mitochondrial encephalomyopathies are caused primarily by point mutations in mt RNAs: mitochondrial encephalopathy, lactic acidosis and stroke-like syndrome (MELAS), caused by a mutation in mt tRNA^Leu(UUR)^; and myoclonus epilepsy with ragged-red fibers (MERRF), caused by a mutation in mt tRNA^Lys^. Approximately 80% of MELAS patients have an A-to-G mutation at position 3243 (A3243G) (16) in the mt tRNA^Leu(UUR)^ gene, and another 10% have a T-to-C mutation at position 3271 (17). MERRF patients have an A8344G mutation in the mt tRNA^Lys^ gene (18). We previously reported that τm^5^U and τm^5^s^2^U were not present in mutant mt tRNA^Leu(UUR)^ harboring the A3243G or T3271C mutation (19), or in mt tRNA^Lys^ harboring the A8344G mutation (20). We also confirmed the absence of taurine modifications in MELAS patient tissues harboring A3243G, G3244A, T3258C, T3271C or T3291C mutations (21), as well as in MERRF patients harboring the A8344G mutation (22). These pathogenic point mutations are assumed to act as negative determinants of τm^5^(s^2^)U biogenesis. In MELAS, the absence of τm^5^U in mt tRNA^Leu(UUR)^ results in a defect in decoding the UUG codon, leading to lower expression of the UUG-rich protein ND6 (23). Similarly, in MERRF the absence of both 5-taurinomethyl and 2-thio groups of τm^5^s^2^U in mt tRNA^Lys^ leads to severe translation failure of both types of AAR codon (24). These observations imply that deficiency in modification of mt tRNA plays a key role in molecular pathogenesis. As mentioned earlier, unmodified U34 can read any of four bases at the third letter of codons in a family box by the four-way wobbling. However, unmodified U34 is only used in tRNAs responsible for family box codons in which at least one G or C is present at the first or second letter of codons. If the codon–anticodon interaction is stabilized by one or two GC pairing at the first two base pairings, U34 is considered to read any of four bases in the family boxes. In the case of mt tRNA^Leu(UUR)^ and mt tRNA^Lys^, there is no G or C at the first or second letter of their cognate codons. This is the reason why unmodified U34 in the mutant tRNAs cannot decipher cognate codons efficiently, not by expanding their decoding capacity.

Mitochondrial diseases are also caused by pathogenic mutations in nuclear-encoded genes (2), including genes encoding translation factors, aminoacyl-tRNA synthetases, tRNA processing enzymes and tRNA-modifying enzymes. Loss-of-function mutations in these genes hamper the biogenesis and function of mt tRNAs. Several instances of pathogenic point mutants in tRNA-modifying enzymes have been reported to date: mitochondrial myopathy and sideroblastic anemia (MLASA), acute infantile liver failure and hypertrophic cardiomyopathy with lactic acidosis are associated with pathogenic mutations in *PUS1* (25), *MTU1* (26) and *MTO1* (27,28), respectively.

To gain more insight into the molecular basis of the mitochondrial decoding system, as well as the molecular pathogenesis of human diseases caused by deficiencies in mt tRNA modifications, it is necessary to obtain a complete picture of post-transcriptional modifications in all 22 species of mammalian mt tRNAs. To date, 11 species of human or bovine mt tRNAs have been sequenced and their post-transcriptional modifications determined (2). In previous work, we isolated all 22 species of bovine mt tRNAs (29). By analyzing these tRNAs by mass spectrometry, we determined the post-transcriptional modifications of seven species of mitochondrial tRNAs that have never been reported, and provide some revised information regarding the modified bases in five other mitochondrial tRNAs. In total, we identified 15 species of modified nucleosides at 118 positions in 22 species of bovine mt tRNAs. We discuss the basic principles of the mitochondrial decoding system in mammals, and propose candidate tRNA-modifying enzymes whose roles remain to be confirmed experimentally.

## MATERIALS AND METHODS

### Isolation of individual mitochondrial tRNAs from bovine liver

Bovine liver RNA was prepared as described previously (6,29). Briefly, crude RNA was extracted from buffer-homogenized bovine liver by phenol extraction, and the tRNA fraction was roughly concentrated by anion exchange chromatography. Individual tRNAs were isolated by chaplet column chromatography (29). Seventeen of them were isolated homogeneously based on the polyacrylamide gel electrophoresis analysis. Five mt tRNAs for Leu(UUR), Asn, Thr, Met and Pro were isolated as major bands but had some minor bands. However, the qualities of the isolated tRNAs are sufficient enough to analyze post-transcriptional modifications.

### Cyanoethylation of pseudouridine in tRNA

Cyanoethylation of tRNA was performed basically as described (30). Eight micrograms of isolated tRNA dissolved in less than 4 μl of Milli-Q water was added to 30 μl of 41% (v/v) ethanol/1.1 M trimethylammonium acetate (pH 8.6). After addition of 4 μl of acrylonitrile (Wako Pure Chemical Industries), the mixture was incubated at 70°C for 2 h, lyophilized and dissolved in Milli-Q water. The solution was then subjected to RNase digestion and analyzed by RNA mass spectrometry, as described below.

### RNA mass spectrometry

RNA fragments digested by RNases were analyzed by mass spectrometry as described previously (31,32). In brief, 2–5 ng of isolated tRNA was digested with RNase T_1_ (Epicentre) or RNase A (Ambion) and analyzed by an LTQ Orbitrap mass spectrometer (Thermo Scientific) with a nano-electrosprayer connected with a splitless nanoflow high pressure liquid chromatography system (DiNa, KYA Technologies). Alternatively, 0.4–2 μg isolated tRNA was digested with RNase T_1_ and analyzed on a LCQ DUO ion-trap mass spectrometer with an ESI (electrospray ionization) source (Thermo Finnigan) and HP1100 liquid chromatography system (Agilent Technologies) in negative ion detection mode. Nucleoside analysis was performed as described previously (31). In brief, about 4 μg of isolated tRNA was digested to nucleosides with nuclease P1 (Wako Pure Chemical Industries) and bacterial alkaline phosphatase C75 (Takara bio), and then analyzed on the LCQ DUO ion-trap mass spectrometer with an ESI source and HP1100 liquid chromatography system in positive-ion detection mode. ProMass (Novatia) was used to obtain the uncharged masses of whole tRNAs by deconvolution of the multiply charged mass spectra.

## RESULTS

### Isolation of 22 species of bovine mt tRNAs

To date, 11 species of bovine mt tRNAs have been described in published work, along with the details of their post-transcriptional modifications: Ser(UCN) (33), Ser(AGY) (34,35), Phe (36), Arg (35), Gly (35), Ile (35), Met (8,37), Val (35), Trp (35), Glu (7) and Gln (7). In addition, sequences of four species of bovine mt tRNAs for Thr, Leu(UUR), Leu(CUN) and Lys have recently been deposited in the tRNA databases as personal communications (38,39). However, some tRNA sequences deposited in the tRNA databases contain unidentified modified bases and mis-annotation of modifications determined by outmoded methods. To accurately define the complete landscape of post-transcriptional modifications of mammalian mt tRNAs, we analyzed all 22 species of mt tRNAs, which we previously isolated from bovine liver by chaplet column chromatography (29).

### Mass spectrometric analysis of post-transcriptional modifications in bovine mt tRNAs

**Figure 1.**
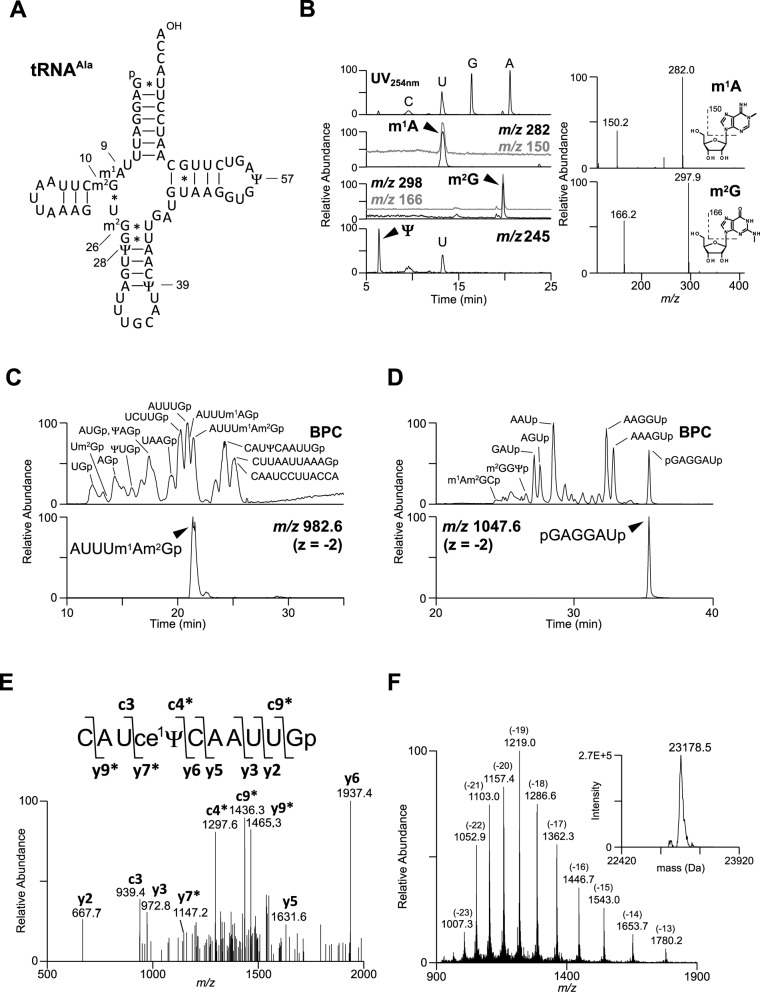
Mass spectrometric analysis of bovine mt tRNA^Ala^ for assignment of post-transcriptional modifications. (A) Secondary structure of bovine mitochondrial tRNA^Ala^ with post-transcriptional modifications determined in this study. The position numbers of the modifications are displayed according to the nucleotide numbering system from the tRNA compilation (40). Symbols for modified nucleosides are as follows: m^1^A, 1-methyladenosine; m^2^G*, N*^2^-methylguanosine and Ψ, pseudouridine. Watson–Crick base pairs are indicated by solid lines, whereas G–U pairs are indicated by asterisks. (B) Nucleoside analysis of bovine mitochondrial tRNA^Ala^. Left, top panel: UV chromatogram at 254 nm of the four major nucleosides (C, U, G and A). Left, lower panels: extracted-ion chromatograms (XIC) for the protonated ion of m^1^A nucleoside (*m/z* 282, black line) with its base ion (*m/z* 150, gray line) (second panel), m^2^G nucleoside (*m/z* 298, black line) with its base ion (*m/z* 166, gray line) (third panel) and Ψ nucleoside (*m/z* 245, black line) (bottom panel). The XIC for the base ion (20% upper offset) is overlaid on the XIC for the nucleoside ion. Right: mass spectra of m^1^A and m^2^G. Cleavage positions for the base-related ions are indicated on the chemical structures. (C) RNA fragment analysis of RNase T_1_ digests of bovine mitochondrial tRNA^Ala^. Assigned fragments are indicated on the base peak chromatogram (BPC) in the first panel. ‘p’ stands for the terminal phosphate group. The XIC for the doubly charged negative ion of a modification-containing fragment (AUUUm^1^Am^2^Gp, *m/z* 982.6) is indicated in the second panel. Because m^2^G at position 10 is a partial modification, both AUUUm^1^Am^2^Gp and AUUUm^1^AGp were detected. (D) RNA fragment analysis of RNase A digests of bovine mitochondrial tRNA^Ala^. Assigned fragments are indicated on the BPC. The XIC for the doubly charged negative ion of the 5′-terminal fragment (pGAGGAUp, *m/z* 1047.6) is indicated in the second panel. (E) A CID spectrum of a cyanoethylated RNA fragment to determine the location of a Ψ site. The doubly charged negative ion of the RNA fragment (*m/z* 1617.7) shown in the inset was used as the precursor ion for CID. The product ions were assigned according to McLuckey *et al.* (41). The asterisks in the spectrum denote product ions containing ce^1^Ψ. (F) Whole mass analysis of intact bovine mt tRNA^Ala^. A series of multiply charged negative ions is shown in the mass spectrum. The charge values are indicated in parentheses. The observed mass obtained by deconvoluting the mass spectra is shown in the inset.

We performed a series of mass spectrometric analyses of each individual mt tRNA to determine its post-transcriptional modifications. As an example, determination of post-transcriptional modifications in mt tRNA^Ala^ is depicted in Figure 1A. First, nucleoside analysis by LC/ESI-MS was carried out to determine the composition of modifications, revealing that 1-methyladenosine (m^1^A), *N*^2^-methylguanosine (m^2^G) and pseudouridine (Ψ) were present in mt tRNA^Ala^ (Figure 1B). In parallel, we used capillary LC/nano ESI-MS to analyze RNA fragments generated by digestion with RNase T_1_ (Figure 1C) or RNase A (Figure 1D). Unmodified fragments were identified by comparing the observed *m/z* values with the calculated *m/z* values deduced from the mt tRNA^Ala^ gene encoded in bovine mtDNA (GenBank accession number V00654). Nucleotide compositions of internal fragments were calculated as 5′-hydroxyl (5′OH) and 3′-phosphate (3′p). The sequence of each fragment was further analyzed by collision-induced dissociation (CID) (Figure 1E). Because tRNA has 5′-phosphate and 3′-hydroxyl groups, the 5′-terminal fragment pGAGGAUp (MW 2097.2) and the 3′ terminal fragment _OH_-CAAUCCUUACCA-_OH_ (MW 3697.5) were detected as species with unique molecular masses distinct from those of the internal fragments. Furthermore, RNA fragments containing modified nucleosides could be identified from the deduced molecular mass calculated from the combinations of unmodified and expected modified nucleotides. For example, in the RNase T_1_ digest of mt tRNA^Ala^, we found a species with *m/z* 982.627, corresponding to a doubly charged negative ion of dimethylated AUUUAGp. The positions of modifications were determined by CID analysis, which indicated the modified sequence AUUUA(+me)G(+me)p. According to the nucleoside analysis, this species was assigned as AUUUm^1^Am^2^Gp, with m^1^A and m^2^G at positions 9 and 10, respectively. Similarly, m^2^G was also found at position 26.

To identify Ψ, a mass-silent modification, each tRNA was treated with acrylonitrile to cyanoethylate Ψ (1-cyanoethyl Ψ; ce^1^Ψ). The derivatized tRNA was then digested by RNase T_1_ and subjected to mass spectrometry to detect the cyanoethylated fragments with molecular mass increased by 53 Da. Three cyanoethylated fragments were detected in the RNase T_1_ digest, and each fragment was further analyzed by CID. As shown in Figure 1E, Ψ at position 39 was identified from the assignment of product ions of CAUce^1^ΨCAAUUGp. Accordingly, we identified three Ψs at positions 28, 39 and 57. Finally, to confirm the assignment of post-transcriptional modifications, the total molecular mass of mt tRNA^Ala^ was measured by deconvoluting the multiply charged negative ions produced by ESI (Figure 1F). The observed mass (23178.5 Da) was fairly close to the calculated mass (23180.6 Da), which corresponds to the base composition of mt tRNA^Ala^ (pU_27_C_9_A_20_G_16_) with three methyl groups. Other tRNAs were basically analyzed using the same procedure as for mt tRNA^Ala^ (Supplementary Information and Table S1).

### Post-transcriptional modifications in seven bovine mt tRNAs

For 10 of the species of mt tRNAs described in the previous literature, our determinations of modifications yielded results consistent with published findings: Phe, Gly, Leu(CUN), Met, Arg, Ser(UCN), Ser(AGY), Val, Glu and Gln (Supplementary Figure S1).

**Figure 2.**
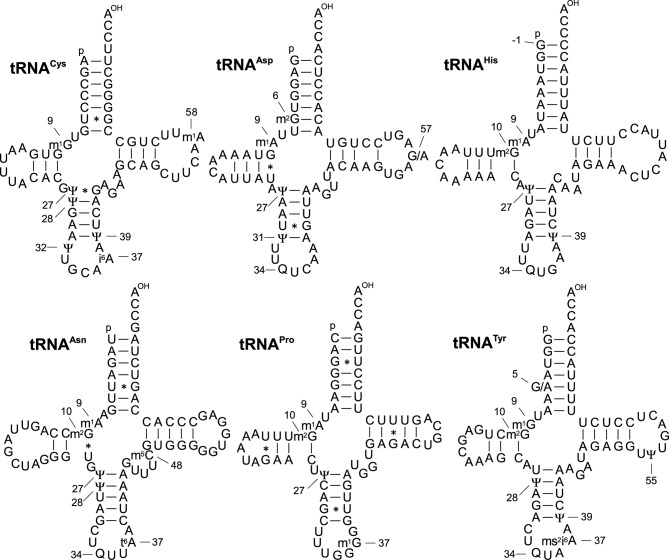
Post-transcriptional modifications in six bovine mt tRNAs. Symbols for modified nucleosides are as follows: m^1^G, 1-methylguanosine; i^6^A, *N^6^*-isopentenyladenosine; Q, queuosine; m^5^C, 5-methylcytidine; ms^2^i^6^A, 2-methylthio-*N^6^*-isopentenyladenosine. The ‘G/A’ in tRNA^Asp^ and tRNA^Tyr^ indicates that both G and A were detected at this position, probably as a result of heteroplasmy in the bovine liver we used in this study.

Several of the tRNAs we characterized have not been previously reported or deposited in any databases. We determined the primary structures and modifications for seven such mt tRNAs: Cys, Asp, His, Asn, Pro and Tyr (Figure 2 and Supplementary Table S1) plus the aforementioned mt tRNA^Ala^ (Figure 1). We identified queuosine (Q) at the wobble positions of mt tRNAs for Asp, His, Asn and Tyr. In cytosolic tRNA^Asp^ and tRNA^Tyr^, Q was glycosylated to mannosyl-Q and galactosyl-Q, respectively; however, Q was not glycosylated in the mt tRNAs. The mt tRNAs for Ala and Pro correspond to family boxes; as expected, unmodified U was present at the wobble position. In mt tRNA^His^, we identified a G at position -1 (G_-1_) (Supplementary Figure S2). This base is likely to be added enzymatically after transcription and 5′-end processing. In mt tRNA^Asp^, m^2^G was present at position 6, the first such case to be reported in mammalian mitochondria. At position 37, which is 3′-adjacent to the anticodon, *N^6^*-isopentenyladenosine (i^6^A) and 2-methylthio-*N^6^*-isopentenyladenosine (ms^2^i^6^A) were present in mt tRNA^Cys^ and mt tRNA^Tyr^, respectively. *N^6^*-threonylcarbamoyladenosine (t^6^A) and m^1^G were also present in the mt tRNAs for Asn and Pro, respectively. During our analysis, we found two polymorphisms in the sequences of mt tRNAs for Asp and Tyr: in mt tRNA^Asp^, a mixture of A and G were present at position 57; similarly, A and G were mixed at position 5 in mt tRNA^Tyr^ (Figure 2). Both sites are encoded as A7356 (H-chain) and A5681 (L-chain) in the bovine mtDNA sequence (GenBank V00654) used as a reference. We speculate that these polymorphisms are the result of mtDNA heteroplasmy in the bovine liver we analyzed. In fact, both sites are encoded as guanosines in the mtDNA sequence of *Bos taurus* isolate FC3 (GenBank accession number DQ124389.1), suggesting that these sites are polymorphic in populations of healthy individuals.

### Full complement of modifications in five bovine mt tRNAs

**Figure 3.**
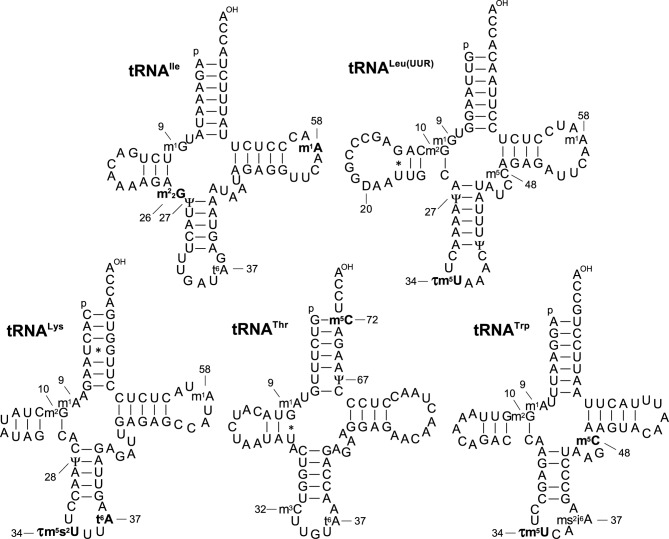
Revised information regarding post-transcriptional modifications of five bovine mt tRNAs. The updated modified bases are represented in bold type. Symbols for modified nucleoside are as follows: m^2^_2_G*, N^2^, N^2^*-dimethylguanosine; D, dihydrouridine; τm^5^U, 5-taurinomethyluridine; τm^5^s^2^U, 5-taurinomethyl-2-thiouridine; t^6^A, *N^6^*-threonylcarbamoyladenosine; m^3^C, 3-methylcytidine.

Although sequences and modifications of the remaining five mt tRNAs [Ile, Leu(UUR), Lys, Thr and Trp] have been published or deposited in the database (38,39), we discovered eight previously unreported modifications in these tRNAs (Figure 3): *N*^2^, *N*^2^-dimethylguanosine (m^2^_2_G) and m^1^A at positions 26 and 58 in mt tRNA^Ile^; τm^5^U at position 34 in mt tRNAs for Leu(UUR) and Trp; τm^5^s^2^U and t^6^A at positions 34 and 37 in mt tRNA^Lys^ and 5-methylcytidine (m^5^C) at position 72 in mt tRNA^Thr^ and position 48 in mt tRNA^Trp^. The wobble modifications of bovine mt tRNAs for Leu(UUR), Lys and Trp remained unidentified in tRNAdb (38). In a previous study (6), we reported that τm^5^U and τm^5^s^2^U were present in bovine mt tRNA^Leu(UUR)^ and mt tRNA^Lys^, respectively. To date, however, their full sequences had not been reported. The m^5^C72 in mt tRNA^Thr^ is the first reported instance of this modification in mammalian mitochondria.

## DISCUSSION

**Figure 4.**
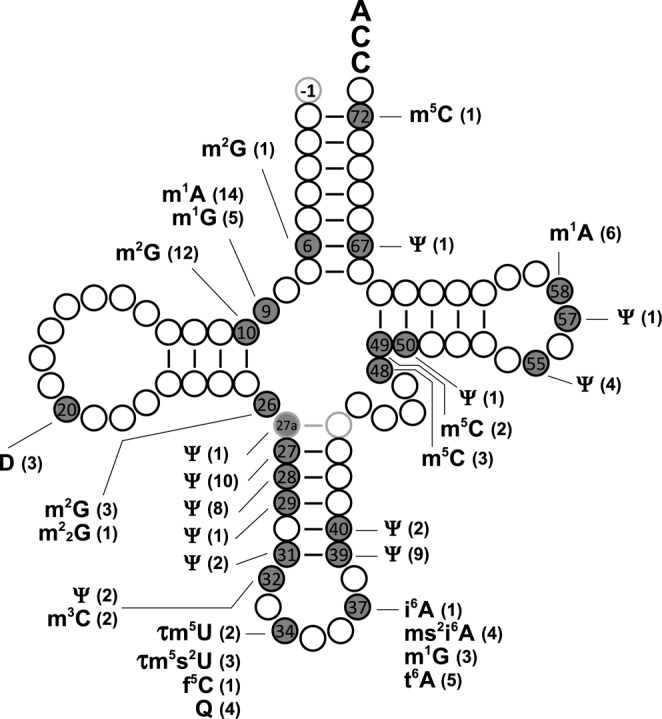
Summary of post-transcriptional modifications in bovine mt tRNAs. Species and numbers of post-transcriptional modifications identified in 22 bovine mt tRNAs are mapped on the schematic secondary structure of tRNA. The modified positions are depicted by gray circles with a symbol indicating each modification. At each position, the number of tRNAs that bear the modification are shown in parenthesis. Positions 27a and 43a, indicated by light gray circles, are unique to mt tRNA^Ser(UCN)^. G_-1_ is specific to mt tRNA^His^.

Here, we report the post-transcriptional modifications in seven species of bovine mt tRNAs not previously characterized, and eight previously unidentified modified bases in five mt tRNAs whose sequences and modifications were determined in earlier studies. In total, we identified 15 species of modified nucleosides at 118 positions in the complete set of bovine mt tRNAs (Supplementary Table S2), i.e. 7.48% of the bases in these tRNAs are modified. The sites and species of all modifications are summarized in a schematic cloverleaf structure (Figure 4). Notably, all modifications are base modifications; the absence of 2′-*O*-methylation is a characteristic feature of mt tRNAs.

Eight mt tRNAs responsible for family boxes had unmodified U at their wobble positions (Table 1), suggesting that the family boxes in mitochondria are decoded by single tRNAs via the four-way wobble rule. For NNR codons, all six mt tRNAs had wobble modifications (Table 1): f^5^C in mt tRNA^Met^, τm^5^U in mt tRNAs for Leu(UUR) and Trp and τm^5^s^2^U in mt tRNAs for Lys, Glu and Gln. We consider that these modifications are required for efficient recognition of the cognate codons, as well as to prevent misreading of near-cognate codons. Q was present at the wobble position in four mt tRNAs (Tyr, His, Asn and Asp) responsible for NAY codons (Table 1). Q34 is known to restrict the conformational flexibility of the anticodon loop by making hydrogen bonds between the side chain amine of Q-base and 2′ OH of U33 (42). Q34 enables tRNA to decode NAU efficiently (43). Although the functional role of Q still remains obscure, it is associated with various physiological events in cytoplasmic tRNAs, including cell development and proliferation, neoplastic transformation and translational read-through or frameshift essential for retroviral production (44–46). These facts suggest that the presence of Q in the four mt tRNAs plays a modulatory role in deciphering NAY codons.

Other mt tRNAs responsible for NNY codons [Phe, Ile, Cys, Ser(AGY)] had unmodified G at the wobble position (Table 1). The results clearly reveal a general principle to decipher the minimal decoding system by the base modifications at the wobble positions.

Hypermodifications are frequently found at position 37 of tRNAs. These modifications play important roles in maintaining translational efficiency and integrity (47,48). Four species of base modifications were present at position 37 in 13 mt tRNAs (Figure 4 and Supplementary Figure S1). Among six mt tRNAs responsible for UNN codons, five had i^6^A or ms^2^i^6^A at position 37. t^6^A37 was present in the mt tRNAs for Ile, Thr, Asn, Lys and Ser(AGY), whereas m^1^G37 was present in the mt tRNAs for Leu(CUN), Pro and Gln. Last year, we discovered cyclic t^6^A, which is formed by ATP-dependent dehydration of t^6^A catalyzed by TcdA, as a bonafide modification at position 37 of tRNAs from bacteria, yeast, plants and protists (49). However, we did not detect any cyclic form of t^6^A in bovine mt tRNAs, and there is no homolog of TcdA in mammalian genomes.

Loss-of-function mutations in tRNA-modifying enzymes can cause human diseases. Consistent with this idea, large-scale disease-associated exome analyses have identified a number of genes that encode tRNA-modifying enzymes (50,51). Identification of all genes involved in mt tRNA modifications will help us to identify genes and mutations associated with various diseases, especially those linked to mitochondrial dysfunction. Basically, the structure and sequence of human mt tRNAs are similar to those of bovine mt tRNAs. In fact, 81% of total bases in 22 mt tRNAs are conserved between these two mammals. That is why human mt tRNA-modifying enzymes can be predicted based on information of bovine mt tRNAs. So far, nine human genes have been confirmed to be responsible for base modifications in mammalian mt tRNAs, and others have been predicted based on studies of tRNA modifications in model organisms (Table 2). Several of these modifications, and the enzymes that may catalyze their formation, are described in the following paragraphs.

**Table 2.** List of confirmed and predicted genes responsible for post-transcriptional modifications in mammalian mt tRNAs

Position ^a^	tRNA species	Modification ^b^	Confirmed gene(s) in human or mammals	Predicted gene(s) in human
6	Asp	m^2^G		*THUMPD3* or *THUMPD2* (52,53)
9	Ala, Asp, Glu, Phe, Gly, His, Lys, Leu(CUN),Asn, Pro, Arg, Thr, Val, Trp	m^1^A	*TRMT10C* and *SDR5C1* (54)	
	Cys, Ile, Leu(UUR), Gln, Tyr	m^1^G	*TRMT10C* and *SDR5C1* (54)	
10	Ala, Phe, Gly, His, Lys, Leu(UUR), Leu(CUN), Asn, Pro, Val, Trp, Tyr	m^2^G		*TRMT11* and *TRMT112* (55)
20	Leu(UUR), Leu(CUN), Ser(UCN)	D		*DUS2* (56)
26	Ala, Glu, Leu(UUR)	m^2^G		*TRMT1*
	Ile	m^2^_2_G		*TRMT1* (57,58)
27a ^c^	Ser(UCN)	Ψ		*PUS1*
27	Cys, Asp, His, Ile, Leu(UUR), Leu(CUN), Asn, Pro, Val, Met	Ψ	*PUS1* (25)	
28	Ala, Cys, Glu, Lys, Leu(CUN), Asn, Ser(UCN), Tyr	Ψ	*PUS1* (25)	
29	Ser(UCN)	Ψ		*PUS1*
31	Asp, Leu(CUN)	Ψ		*RPUSD1*, *RPUSD2*, *RPUSD3* or *RPUSD4*^d^
32	Cys, Val	Ψ		*RPUSD1*, *RPUSD2*, *RPUSD3* or *RPUSD4*^d^
	Ser(UCN), Thr	m^3^C		*METTL2B* (59,60)
34	Leu(UUR), Trp, Glu, Lys, Gln,	τm^5^U		*GTPBP3* and *MTO1* (61,62)
	Glu, Lys, Gln	τm^5^s^2^U ^e^	*MTU1*^e^ (63) and *NFS1*^e^ (64)	
	Met	f^5^C		Unidentified
	Asp, His, Asn, Tyr	Q		*hQTRT1* and *hQTRTD1* (65,66)
37	Ile, Lys, Asn, Ser(AGY), Thr	t^6^A		*YRDC* and *QRI7* (*OSGEPL1*) (67)
	Cys, Phe, Ser(UCN), Trp, Tyr	i^6^A	*TRIT1* (68)	
	Phe, Ser(UCN), Trp, Tyr	ms^2^i^6^A^f^	*CDK5RAP1*^f^ (69)	
	Leu(CUN), Pro, Gln	m^1^G	*TRMT5* (70)	
39	Ala, Cys, Phe, Gly, His, Leu(UUR), Gln, Arg, Tyr	Ψ		*PUS3* (71)
40	Glu, Gln	Ψ		*PUS3*^d^
48	Leu(UUR), Asn, Trp	m^5^C		*NSUN2* (72,73)
49	Glu, Ser(AGY)	m^5^C		*NSUN2* (72,73)
50	Met	Ψ		Unidentified
55	Glu, Gln, Ser(UCN), Tyr	Ψ		*TRUB2* (74)
57	Ala	Ψ		Unidentified
58	Cys, Glu, Ile, Lys, Leu(UUR), Ser(UCN)	m^1^A	*TRMT61B* (75)	
67	Thr	Ψ		*PUS1*^d^ (76)
72	Thr	m^5^C		Unidentified

^a^Numbering system for tRNA comes from the tRNAdb compilation (40).

^b^Symbols for modifications originate from MODOMICS (http://modomics.genesilico.pl/) (39).

^c^This position number is unique to mt tRNA^Ser(UCN)^ (77).

^d^These predictions were altered from our previous prediction (2).

^e^MTU1 and NFS1 are involved in 2-thiolation of τm^5^s^2^U34. *MTU1* (Mitochondrial tRNA-specific 2-thiouridylase 1) is known as *TRMU* which originated from bacterial *trmU* (tRNA methyltransferase U). However, *trmU* (renamed as *mnmA*) was found to be a mis-annotation because it is not a tRNA methyltransferase.

^f^CDK5RAP1 is required for 2-methylthiolation of ms^2^i^6^A37.

On the basis of studies of bacterial and yeast mitochondria (63,78), GTPBP3 and MTO1 are predicted to be enzymes involved in τm^5^U formation; however, to date, no direct evidence in support of this prediction has been published. The GTPBP3–MTO1 complex has been proposed to recognize mt tRNAs for Leu(UUR), Trp, Lys, Glu and Glu. However, these five mt tRNAs share little sequence similarity and no consensus motif. Nonetheless, τm^5^U formation is sensitive to a single pathogenic point mutation associated with MELAS or MERRF (12), implying strict substrate specificity of the enzyme. To resolve this issue, it will be necessary to perform *in vitro* reconstitution of τm^5^U on mt tRNAs using recombinant proteins.

We previously identified MTU1, a thiouridylase responsible for 2-thiolation of τm^5^s^2^U in mt tRNAs (63). The sulfur atom comes from Cys, a process mediated by a cysteine desulfurase, NFS1 (64). During bacterial 2-thiouridine formation, several sulfur mediators transfer persulfide sulfur from cysteine desulfurase to thiouridylase (79). To date, however, there is no information available regarding the involvement of such sulfur mediators in the formation of 2-thiouridine in mitochondria.

In mammals, the Q-base is obtained either from the diet or through intestinal microflora (45), because no homologous genes responsible for Q-base biogenesis *de novo* are encoded in mammalian genome. Mammals possess two tRNA-guanine transglycosylases (TGTases), QTRT1 and QTRTD1, which may transglycosylate the wobble base with the dietary Q-base. According to its subcellular localization, QTRTD1 appears to be the mitochondrial TGTase (65,66). Further studies will be necessary to confirm the identity of the mitochondrial TGTase, as well as the transport pathway by which dietary Q enters the mitochondria. Q is not present in mt tRNA^Asp^ from Morris hepatoma cells (80).

In yeast, t^6^A37 is synthesized by two enzymes, Sua5p and Qri7p (67). YRDC and OSGEPL1, the human homologs of yeast Sua5p and Qri7p, are presumably involved in t^6^A formation in mammalian mt tRNAs.

*TRIT1* is a tumor suppressor gene, mutations in which are associated with cancer progression. Human TRIT1 was identified as a tRNA isopentenyltransferase (IPTase) homologous to bacterial MiaA and yeast Mod5; the latter is responsible for i^6^A37 formation in both cytoplasmic and mt tRNAs. Knockdown of TRIT1 in human cells reduced the abundance of i^6^A in mt tRNA^Ser(UCN)^ (68). In the present study, we determined that bovine mt tRNAs for Tyr and Cys have i^6^A37; in total, five mt tRNAs have been identified as potential substrates for TRIT1 in mitochondria.

The human protein CDK5RAP1 is a 2-methylthiolase that is homologous to bacterial MiaB. A CDK5RAP1 variant localizes in mitochondria, and knockdown of CDK5RAP1 results in a reduction of ms^2^i^6^A levels (69). Thus, four mt tRNAs [Phe, Ser(UCN), Tyr and Trp] are likely to be modified by CDK5RAP1.

TRMT5, a methyltransferase responsible for m^1^G37 formation, is predicted to be localized to the mitochondria (70,81), and recombinant TRMT5 can modify *in vitro* transcribed mt tRNA (70), suggesting that TRMT5 methylates three mt tRNAs: Leu(CUN), Pro and Gln.

Base methylation at position 9 is frequently observed among bovine mt tRNAs. Indeed, 19 mt tRNAs have m^1^A9 or m^1^G9 (Figure 4). m^1^A9 stabilizes the canonical cloverleaf structure of mt tRNAs (82). In addition, m^1^A9 is indispensable for aminoacylation of nematode mt tRNAs that lack the T-arm. The methyltransferase for m^1^A9 or m^1^G9 has been identified as a complex of TRM10C and SDR5C1, both of which are components of mt RNase P (54). The TRM10C–SDR5C1 complex has broad substrate specificity for 19 out of 22 mt tRNA species. Only three mt tRNAs are unmethylated at position 9. Of these, mt tRNA^Met^ has C at this position, and the two mt tRNA^Ser^ isoacceptors have non-canonical cloverleaf structures: Type I for mt tRNA^Ser(UCN)^ and Type III for mt tRNA^Ser(AGY)^ (2).

Ψ is the most abundant modification in bovine mt tRNAs. Among the 22 mt tRNAs, we mapped Ψ at 42 sites, 35 of which reside in the anticodon arm. In humans, there are 13 species of Ψ synthases, each of which has different substrate specificity and introduces Ψ at multiple sites in various RNAs. PUS1 is a human Ψ synthase with broad substrate specificity that introduces Ψ into cytoplasmic tRNAs, mt tRNAs, U2 snRNA and SRA non-coding RNA (25,76,^83–84^), and it is responsible for Ψ27 and Ψ28 in mt tRNAs (25). We assume that PUS1 is also responsible for Ψ27a and Ψ29 in mt tRNA^Ser(UCN)^ and Ψ67 in mt tRNA^Thr^ (76). Because missense mutations in the PUS1 gene are responsible for the development of MLASA (25), it will be necessary to determine the Ψ sites targeted by PUS1 to gain a deeper understanding of the functional role of this modification, as well as the molecular pathogenesis of MLASA. According to the studies of yeast and *Escherichia coli* tRNAs, Ψ39 and Ψ40 are likely to be introduced by PUS3 (71), whereas Ψ55 is probably modified by TRUB2 (74). In yeast mitochondria, Ψ31 and Ψ32 are introduced by PUS6 and PUS9, respectively (85,86). Although we previously predicted that either RPUSD2 or RPUSD4 was responsible for Ψ31 and Ψ32 (2), two other homologs, RPUSD1 and RPUSD3, should also be regarded as candidates for these modifications. Regarding Ψ50 and Ψ57, no candidate enzymes can be predicted at present. Biochemical and genetic analyses of Ψ synthase genes will be necessary to determine the enzyme responsible for each Ψ site.

In contrast to cytoplasmic tRNAs, most mammalian mt tRNAs do not have consensus sequences of D- and T-loops, which are conserved among tRNAs in general; consequently, they have lost the canonical D-loop/T-loop interaction. This feature is characteristic of mt tRNAs in mammals (2). In the D-loop, dihydrouridines (D) at positions 16 and 17, which are frequently observed in canonical tRNAs from various sources, are not present in bovine mt tRNAs. Instead, D20 was present in three species of bovine mt tRNAs. Based on a study on yeast tRNA, it is reasonable to predict that human tRNA-dihydrouridine synthase 2 (DUS2) introduces D20 (56). Human DUS2 has been implicated in pulmonary carcinogenesis (87). In the T-loop, Ψ55 and m^1^A58, both of which are typical modifications in canonical tRNAs, are present in four and six mt tRNAs, respectively. We previously identified human TRMT61B as the methyltransferase responsible for m^1^A58 in mt tRNAs for Leu(UUR), Ser(UCN) and Lys (75). In addition to these three tRNAs, we also found m^1^A58 in mt tRNAs for Cys, Glu and Ile. 5-methyluridine (m^5^U) at position 54 is one of the most common T-loop modifications in canonical tRNAs. m^5^U54 is present in the human mt tRNAs for Leu(UUR) (19) and Ser(UCN) (88); however, we did not detect m^5^U54 in any bovine mt tRNAs.

m^2^G6 is present in bovine mt tRNA^Asp^. Human mt tRNA^Asp^ does not have m^2^G6 (89), because this position is replaced by A6. The methyltransferase responsible for m^2^G6 was identified as Trm14 in *Methanocaldococcus jannaschii* (52) and TTH1157 in *Thermus thermophilus* (53). A similarity search using the Trm14 sequence retrieved two human homologs, THUMPD2 and THUMPD3. According to the WoLF P-sort (90) prediction of subcellular localization, the former is likely localized in the cytoplasm and the latter in mitochondria. Although further investigation is necessary, THUMPD3 is the most plausible candidate for the enzyme that introduces m^2^G6 in mt tRNA^Asp^, whereas THUMPD2 probably acts on cytoplasmic tRNAs. Although both genes are encoded in the human genome, no m^2^G6 is present in human mt tRNA^Asp^, implying the presence of other substrates for this enzyme in human mitochondria.

In this study, we identified G_-1_ at the 5′ terminus of bovine mt tRNA^His^. In general, G_-1_ is an identity element for aminoacylation by histidyl-tRNA synthetase (91). This is the first reported instance of this base in mammalian mitochondria, although G_-1_ is present in both yeast and starfish mt tRNA^His^ (92,93). Judging from the fact that the 5′-adjacent nucleotide of the mt tRNA^His^ gene in bovine mt DNA is T rather than G (e.g. GenBank V00654), it is likely that G_-1_ is added enzymatically after 5′-terminal processing by mt RNase P. THG1L is a mammalian guanylyltransferase that adds G_-1_ at the 5′ terminus of cytoplasmic tRNA^His^. Because THG1L is predicted to have a mitochondria-targeting sequence at the N-terminus (94), THG1L is likely to be responsible for G_-1_ addition of mt tRNA^His^.

In summary, we have compiled the first complete picture of post-transcriptional modifications in mammalian mt tRNAs. The results of this study enable a deeper understanding of the molecular mechanisms underpinning the minimal decoding system in mammalian mitochondria, and should help predict the human tRNA-modifying enzymes responsible for each modification in mt tRNAs. The list of tRNA-modifying enzymes serves as a practical landmark that encourages us to identify all genes responsible for tRNA modifications in mammalian mitochondria, and to further investigate human diseases caused by tRNA modification disorders in mitochondria. An important goal for future efforts is the identification of all modifications in human mt tRNAs.

## SUPPLEMENTARY DATA

Supplementary Data are available at NAR Online.

SUPPLEMENTARY DATA
