# Supplementary material for: A complete landscape of post-transcriptional modifications in mammalian mitochondrial tRNAs
Source: Nucleic Acids Res. 2014 May 15;42(11):7346–57. doi: 10.1093/nar/gku390 (PMC4066797; doi:10.1093/nar/gku390)
Supplement: SUPPLEMENTARY DATA [file supp_42_11_7346__index.html]

A complete landscape of post-transcriptional modifications in mammalian mitochondrial tRNAs — A complete landscape of post-transcriptional modifications in mammalian mitochondrial tRNAs — SUPPLEMENTARY DATA 

# A complete landscape of post-transcriptional modifications in mammalian mitochondrial tRNAs

## SUPPLEMENTARY DATA

**Files in this Data Supplement:**

- SUPPLEMENTARY DATA
- SUPPLEMENTARY DATA
- SUPPLEMENTARY DATA
- SUPPLEMENTARY DATA
