# Supplementary material for: A complete landscape of post-transcriptional modifications in mammalian mitochondrial tRNAs
Source: Nucleic Acids Res. 2014 May 15;42(11):7346–57. doi: 10.1093/nar/gku390 (PMC4066797; doi:10.1093/nar/gku390)
Supplement: SUPPLEMENTARY DATA [file supp_gku390_nar-00825-v-2014-File004.pdf]

Supplementary Figure 1

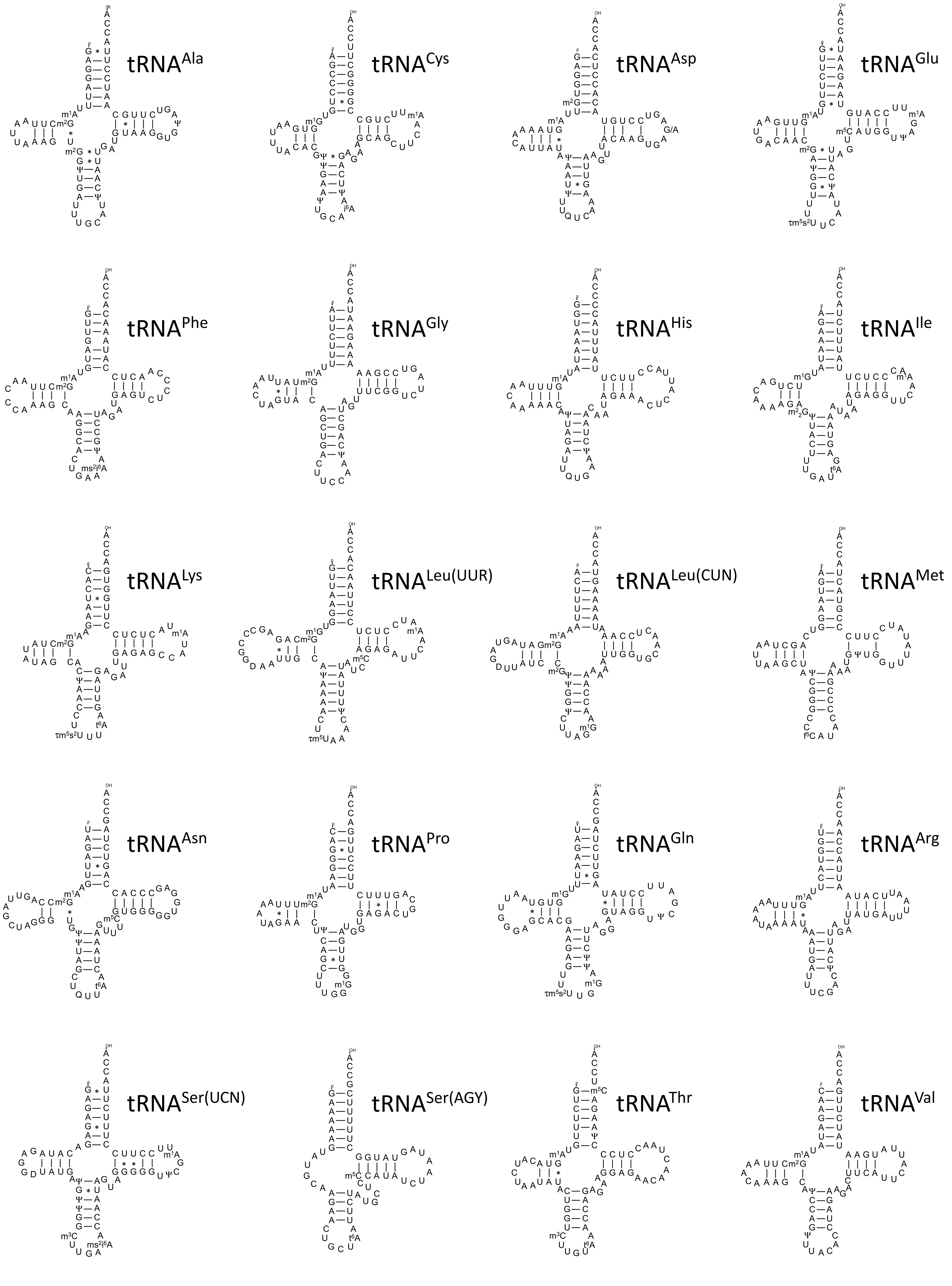

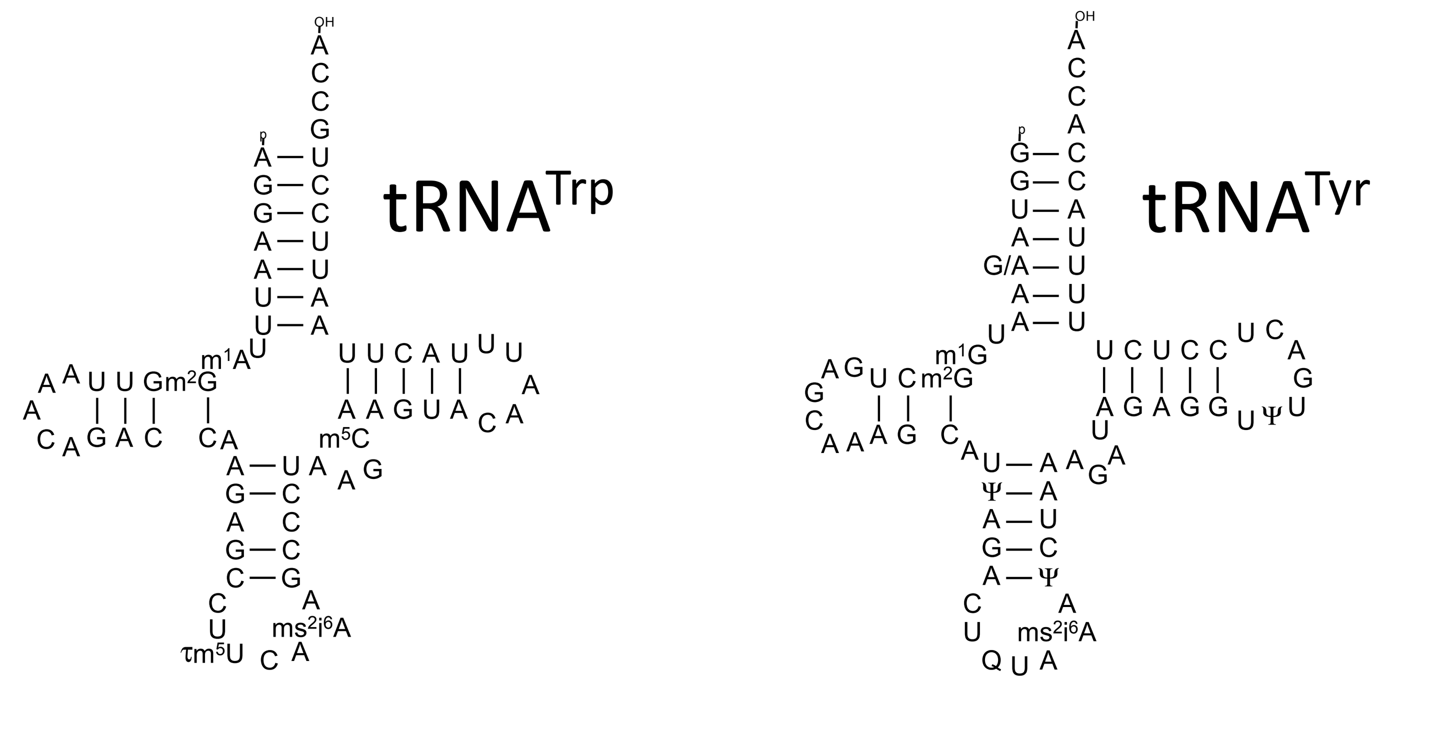

Twenty-two species of bovine mitochondrial tRNAs with post-transcriptional modifications

**m^1A:**	**1-methyladenosine**
**i^6A:**	**N^6-isopentenyladenosine**
**ms^2i^6A:**	**2-methylthio-*N*^{6}-isopentenyladenosine**
**t^6A:**	**N^6-threonylcarbamoyladenosine**
**m^3C:**	**3-methylcytidine**
**m^5C:**	**5-methylcytidine**
**f^5C:**	**5-formylcytidine**
**m^1G:**	**1-methylguanosine**
**m^2G:**	***N*^{2}-methylguanosine**
**$m^2{}_2G$:**	***N*^{2},*N*^{2}-dimethylguanosine**
**Q:**	**queuosine**
**Ψ:**	**pseudouridine**
**D:**	**dihydrouridine**
**τm^5U:**	**5-taurinomethyluridine**
**τm^5s^2U:**	**5-taurinomethyl-2-thiouridine**

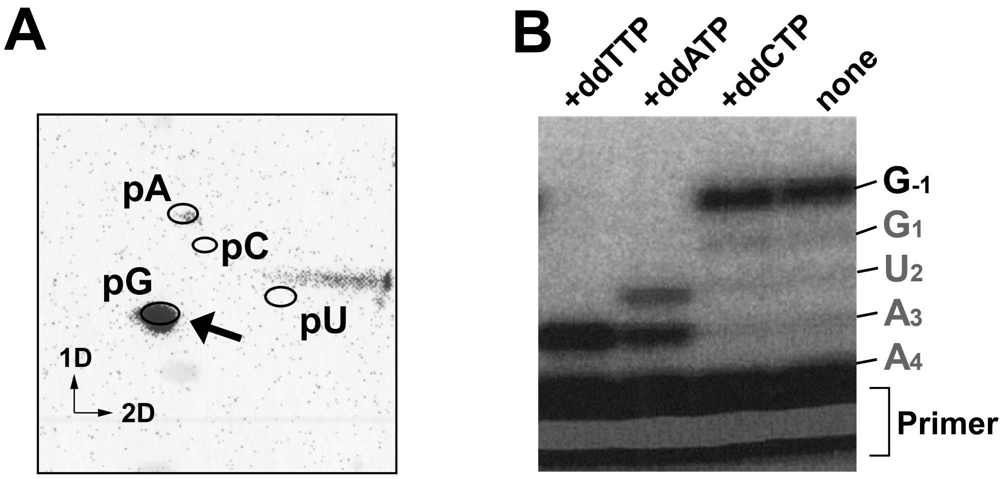

Figure S2
