# Supplementary material for: A complete landscape of post-transcriptional modifications in mammalian mitochondrial tRNAs
Source: Nucleic Acids Res. 2014 May 15;42(11):7346–57. doi: 10.1093/nar/gku390 (PMC4066797; doi:10.1093/nar/gku390)
Supplement: SUPPLEMENTARY DATA [file supp_gku390_nar-00825-v-2014-File003.docx]

**Supplementary Information**

**Supplementary results**

**Determination of post-transcriptional modifications in mt tRNAs**

Six mt tRNAs (Cys, Asp, His, Asn, Pro, and Tyr) which were previously uncharacterized were basically analyzed using the same procedure as for mt tRNA^Ala^. All the modified nucleosides shown in Figure 2 were detected by the nucleotide analysis. In parallel, each tRNA was subjected to the capillary LC/nano ESI-MS analysis of RNA fragments generated by RNase T_1_-digestion for the six tRNAs (Table S1) and RNase A-digestion for three tRNAs (Asp, Asn and Tyr). The site of each modification was determined by assignment of CID spectrum. The Ψ sites in mt tRNAs for Cys, His and Pro were determined by the cyanoethylation analysis. For mt tRNAs for Asp, Asn and Tyr, as the Ψ sites are present in long fragments, we conducted the post-labeling method ([1](#_ENREF_1),[2](#_ENREF_2)) to determine each of Ψ site with other modifications. The G at position -1 in mt tRNA^His^ was confirmed by the 5'-terminal nucleotide analysis and primer extension method (Figure S2). Total molecular mass analysis of each tRNA was consistent with the assignment of post-transcriptional modifications in this analysis. It should be noted here that only m^1^G37 in mt tRNA^Pro^ was exceptionally unable to be determined unambiguously by our current data (i.e. RNase T_1_ analyses). Although we observed m^1^GGp by RNase T_1_ digestion, four possible positions 4, 36, 37 and 38 based on the primary sequence of mt tRNA^Pro^ still remain to be confirmed. However, high conservation of m^1^G37 in most tRNAs^Pro^ strongly supports the presence of m^1^G at position 37 in bovine mt tRNA^Pro^.

**Supplementary methods**

**Post-labeling method to determine the sites of modifications**

The post-labeling method was performed basically as described previously ([1](#_ENREF_1),[2](#_ENREF_2)). In brief, the purified tRNA was partially hydrolyzed by heat treatment. 5' terminus of each fragment in the hydrolysate was ^32^P-phosphorylated. Each labeled fragment was separated by denaturing PAGE, cut out and eluted from the gel, and digested by nuclease P1. The ^32^P-labeled 5'-terminal nucleotide of each fragment was analyzed by two-dimensional thin-layer chromatography (2D-TLC) on cellulose plates. The radiolabeled spots were visualized by an FLA-7000 imaging analyzer (FujiFilm). Each modified nucleotide was determined by referring to the modified nucleotide map.

**5' terminal nucleotide analysis**

About four-ng of 5'-^32^P labeled mt tRNA^His^ was digested in a 10 μL solution containing 1 μg nuclease P1 for 3 hours at 37 °C. One-μL aliquot of the digest was analyzed by 2D-TLC. The following procedure was the same as described above.

**Primer extension for detection of G_-1_ in mt tRNA^His^**

Primer extension was performed basically as described previously ([3](#_ENREF_3)). Two pmol of 5'-^32^P labeled primer (5'-TTTTGTTAAACTATAT-3') was mixed with 80 ng of the isolated mt tRNA^His^ in a 10 μL solution consisting of 10 mM Tris-HCl (pH 8.0) and 1 mM EDTA, incubated at 80 °C for 2 min, and left for several minutes at room temperature. Reverse transcription was carried out at 42 °C for 1h in a 20 μL mixture consisting of the above-mentioned solution, 3.75 mM MgCl_2_, 20 units of M-MLV Reverse Transcriptase RNaseH^-^ (TOYOBO), 1x RTase buffer (supplied with the Reverse Transcriptase, TOYOBO), 37.5 μM each of dNTPs or dNTP/ddNTP mixture. The reaction mixture was subjected to 10% denaturing PAGE. The labeled cDNAs were visualized and analyzed according to a commonly-used procedure.

**Supplementary Tables and Figures**

**Table S1 List of RNase T_1_-digested fragments for seven bovine mt tRNAs**

RNase T_1_-digested RNA fragments longer than dinucleotides and modified dinucleotides with their observed and calculated *m/z* values are listed for bovine mt tRNAs for Ala, Cys, Asp, His, Asn, Pro and Tyr. **a, b, d, e** and **f:** These fragments were generated by partial modifications. **c** and **g:** These fragments originate from polymorphism in tRNA sequences.

**Table S2 Primary sequences of 22 species of bovine mt tRNAs with post-transcriptional modifications**

**Figure S1 Twenty-two species of bovine mt tRNAs with post-transcriptional modifications**

**Figure S2 5'-terminal analysis of mt tRNA^His^**

(A) The ^32^P-labeled 5' terminal nucleotide of mt tRNA^His^, developed on 2D-TLC in solvent system A: isobutyric acid / concentrated ammonia / H_2_O (66 : 1 : 33, v/v/v) in the first dimension, and 2-propanol / HCl / H_2_O (70 : 15 : 15, v/v/v) in the second dimension. Locations of 5' mononucleotide standards (pA, pC, pG and pU) were displayed as circles.

(B) A primer extension of mt tRNA^His^ showed the presence of G at position -1 (+ddCTP and none lanes). The corresponding sequence of 5' terminal region of mt tRNA^His^ was indicated in right side of the gel image.

**REFERENCES**

1. Yasukawa, T., Suzuki, T., Suzuki, T., Ueda, T., Ohta, S. and Watanabe, K. (2000) Modification defect at anticodon wobble nucleotide of mitochondrial tRNAs^Leu^(UUR) with pathogenic mutations of mitochondrial myopathy, encephalopathy, lactic acidosis, and stroke-like episodes. *J Biol Chem*, 275, 4251-4257.

2. Kuchino, Y., Hanyu, N. and Nishimura, S. (1987) Analysis of modified nucleosides and nucleotide sequence of tRNA. *Methods Enzymol*, 155, 379-396.

3. Chujo, T. and Suzuki, T. (2012) Trmt61B is a methyltransferase responsible for 1-methyladenosine at position 58 of human mitochondrial tRNAs. *RNA*, 18, 2269-2276.
