# Supplementary material for: A complete landscape of post-transcriptional modifications in mammalian mitochondrial tRNAs
Source: Nucleic Acids Res. 2014 May 15;42(11):7346–57. doi: 10.1093/nar/gku390 (PMC4066797; doi:10.1093/nar/gku390)
Supplement: SUPPLEMENTARY DATA [file supp_gku390_nar-00825-v-2014-File005.docx]

**Table S1 List of RNase T_1_-digested fragments for seven bovine mt tRNAs**

RNase T_1_-digested RNA fragments longer than dinucleotides and modified dinucleotides with their observed and calculated *m/z* values are listed for bovine mt tRNAs for Ala, Cys, Asp, His, Asn, Pro and Tyr. **a, b, d, e** and **f:** These fragments were generated by partial modifications. **c** and **g:** These fragments originate from polymorphism in tRNA sequences.

| **No.** | **Sequences of RNA fragments** | **Molecular mass** | **Most intense m/z** | | | | **Charge state** |
| --- | --- | --- | --- | --- | --- | --- | --- |
|  |  |  | **observed** | | **calculated** | |  |
|  | *Ala UGC* |  | |  | |  |  |
| **1** | CAAUCCUUACCA (3’ terminus) | 3697.537 | | 1847.9 | | 1847.760 | -2 |
| **2** | CUUAAUUAAAGp | 3537.463 | | 1767.8 | | 1767.724 | -2 |
| **3** | CAUΨCAAUUGp | 3184.399 | | 1591.5 | | 1591.192 | -2 |
| **4** | AUUUm^1^Am^2^Gp | 1967.270 | | 983.0 | | 982.627 | -2 |
| **5 ^a^** | AUUUm^1^AGp | 1953.255 | | 976.0 | | 975.619 | -2 |
| **6** | AUUUGp | 1610.186 | | 804.5 | | 804.085 | -2 |
| **7** | UCUUGp | 1586.175 | | 792.5 | | 792.080 | -2 |
| **8** | UAAGp | 1327.188 | | 1326.2 | | 1326.181 | -1 |
| **9** | ΨAGp | 998.136 | | 997.2 | | 997.128 | -1 |
| **10** | AUGp | 998.136 | | 997.2 | | 997.128 | -1 |
| **11** | ΨUGp | 975.109 | | 974.0 | | 974.101 | -1 |
| **12** | Um^2^Gp | 683.099 | | 682.3 | | 682.091 | -1 |
|  | *Cys GCA* |  | |  | |  |  |
| **1** | CUUCAm^1^AUUCUGp | 3480.429 | | 1739.2 | | 1739.207 | -2 |
| **2 ^b^** | CUUCAAUUCUGp | 3466.413 | | 1732.3 | | 1732.199 | -2 |
| **3** | AAUUUACACGp | 3207.427 | | 1602.9 | | 1602.705 | -2 |
| **4** | CAi^6^AAΨUCAGp | 2969.464 | | 1483.9 | | 1483.724 | -2 |
| **5** | CUUCCA (3’ terminus) | 1794.271 | | 896.4 | | 896.128 | -2 |
| **6** | AAΨUGp | 1633.214 | | 815.9 | | 815.599 | -2 |
| **7** | CCCUGp | 1584.207 | | 791.4 | | 791.096 | -2 |
| **8** | Um^1^GGp | 1028.146 | | 1027.1 | | 1027.139 | -1 |
| **9** | AAGp | 1021.163 | | 1020.1 | | 1020.155 | -1 |
| **10** | CAGp | 997.152 | | 996.2 | | 996.144 | -1 |
| **11** | ΨΨGp | 975.109 | | 974.1 | | 974.101 | -1 |
| **12** | CCGp | 973.141 | | 972.1 | | 972.133 | -1 |
|  | *Asp QUC* |  | |  | |  |  |
| **1** | UAAAACAUUAUAΨAAUΨUUQUCAAAGp | 8453.139 | | 938.7 | | 938.230 | -9 |
| **2** | UACACCUCACCA (3’ terminus) | 3696.553 | | 1231.2 | | 1231.176 | -3 |
| **3** | UUACAAGp | 2267.307 | | 1132.6 | | 1132.646 | -3 |
| **4** | UUAAGp | 1633.214 | | 815.6 | | 815.599 | -2 |
| **5** | UCCUGp | 1585.191 | | 791.6 | | 791.588 | -2 |
| **6 ^c^** | AAAGp | 1350.216 | | 674.1 | | 674.100 | -2 |
| **7** | UUm^1^AGp | 1318.177 | | 658.1 | | 658.081 | -2 |
| **8** | Um^2^Gp | 683.099 | | 682.1 | | 682.091 | -1 |
|  | *His QUG* |  | |  | |  |  |
| **1** | AAACUCAUUACCUUCUUAUUUACCCCA  (3’ terminus) | 8376.089 | | 837.0 | | 836.601 | -10 |
| **2** | UUUAACAAAAACAΨUAGp | 5464.740 | | 1820.4 | | 1820.572 | -3 |
| **3** | AAΨCUAACAAUAGp | 4194.584 | | 1397.9 | | 1397.187 | -3 |
| **4** | UAAAUAUm^1^Am^2^Gp | 2954.428 | | 1475.9 | | 1476.206 | -2 |
| **5 ^d^** | UAAAUAUm^1^AGp | 2940.412 | | 1469.5 | | 1469.198 | -2 |
| **6** | AUUQUGp | 2081.302 | | 1039.5 | | 1039.643 | -2 |
|  | *Asn QUU* |  | |  | |  |  |
| **1** | CUQUUt^6^AACUAAAGp | 4458.657 | | 1485.2 | | 1485.211 | -3 |
| **2** | CCCACCAGp | 2546.369 | | 1272.2 | | 1272.177 | -2 |
| **3** | ΨΨUAGp | 1610.186 | | 804.1 | | 804.085 | -2 |
| **4** | UCUAGp | 1609.202 | | 803.6 | | 803.593 | -2 |
| **5** | UUUm^5^CGp | 1600.191 | | 799.1 | | 799.088 | -2 |
| **6** | UUAGp | 1304.161 | | 651.1 | | 651.073 | -2 |
| **7** | AUUGp | 1304.161 | | 651.1 | | 651.073 | -2 |
| **8** | CUAGp | 1303.177 | | 650.6 | | 650.581 | -2 |
| **9** | CCAGp | 1302.193 | | 650.1 | | 650.089 | -2 |
| **10** | pUAGp (5’ terminus) | 1078.102 | | 1077.1 | | 1077.094 | -1 |
| **11** | Am^1^Am^2^Gp | 1049.194 | | 1048.2 | | 1048.187 | -1 |
| **12 ^e^** | Am^1^AGp | 1035.179 | | 1034.2 | | 1034.171 | -1 |
| **13** | CCA (3’ terminus) | 877.179 | | 876.2 | | 876.172 | -1 |
|  | *Pro UGG* |  | |  | |  |  |
| **1** | UUUCUUCCUUGp | 3420.359 | | 1709.3 | | 1709.172 | -2 |
| **2** | UUUAAAUAGp | 2903.369 | | 1451.0 | | 1450.677 | -2 |
| **3** | AACUΨCAGp | 2572.349 | | 1285.7 | | 1285.167 | -2 |
| **4** | AAUm^1^Am^2^Gp | 1684.272 | | 841.3 | | 841.128 | -2 |
| **5 ^f^** | AAUm1AGp | 1670.257 | | 834.5 | | 834.120 | -2 |
| **6** | CUUUGp | 1586.175 | | 792.3 | | 792.080 | -2 |
| **7** | ACUGp | 1303.177 | | 650.9 | | 650.581 | -2 |
| **8** | ACCA (3’ terminus) | 1206.232 | | 1205.2 | | 1205.224 | -1 |
| **9** | pCAGp (5’ terminus) | 1077.118 | | 1076.2 | | 1076.110 | -1 |
| **10** | AUGp | 998.136 | | 997.2 | | 997.128 | -1 |
| **11** | CAGp | 997.152 | | 996.2 | | 996.144 | -1 |
| **12** | UUGp | 975.109 | | 974.1 | | 974.101 | -1 |
| **13** | m^1^GGp | 722.121 | | 721.2 | | 721.113 | -1 |
|  | *Tyr QUA* |  | |  | |  |  |
| **1** | ACUCCUCUUUUUACCACCA (3’ terminus) | 5836.762 | | 1944.6 | | 1944.579 | -3 |
| **2** | ACUQUAms^2^i^6^AAΨCUAAAGp | 5085.775 | | 725.5 | | 728.587 | -7 |
| **3 ^g^** | UAAAAUm^1^Gm^2^Gp | 2664.397 | | 1331.2 | | 1331.191 | -2 |
| **4** | CAUΨAGp | 1938.255 | | 968.1 | | 968.120 | -2 |
| **5 ^g^** | AAUm^1^Gm^2^Gp | 1700.267 | | 849.1 | | 849.126 | -2 |
| **6** | CAAAGp | 1655.257 | | 826.6 | | 826.621 | -2 |
| **7** | AUAGp | 1327.188 | | 662.6 | | 662.586 | -2 |
| **8** | UΨUGp | 1281.134 | | 639.6 | | 639.559 | -2 |
| **9 ^g^** | UAGp | 998.136 | | 997.1 | | 997.128 | -1 |
| **10** | CUGp | 974.125 | | 973.1 | | 973.117 | -1 |
